# Reproductive and Agronomic Characterization of Novel Apomictic Hybrids of *Paspalum* (Poaceae)

**DOI:** 10.3390/genes14030631

**Published:** 2023-03-02

**Authors:** Elsa Andrea Brugnoli, Alex Leonel Zilli, Florencia Marcón, Enzo Caballero, Eric Javier Martínez, Carlos Alberto Acuña

**Affiliations:** 1 Instituto de Botánica del Nordeste, Facultad de Ciencias Agrarias, Universidad Nacional del Nordeste, Consejo Nacional de Investigaciones Científicas y Técnicas, Corrientes 3400, Argentina; 2 Facultad de Ciencias Agrarias, Universidad Nacional del Nordeste, Corrientes 3400, Argentina

**Keywords:** warm season grasses, apomixis expression, polyploidy, new forages

## Abstract

The tetraploid germplasm of *Paspalum* contains a large diversity that can be used to generate better forages. The objective was to evaluate a group of *Paspalum notatum* and *Paspalum simplex* apomictic hybrids for a set of agronomic traits and apomixis expressivity. Forage yield, cold tolerance, winter regrowth, and seed yield were evaluated. The expressivity of apomixis was evaluated in *P. simplex* hybrids by flow cytometry. Progeny testing with molecular markers was used to determine the genotypic variability in the progeny. Differences within *P. notatum* and *P. simplex* hybrids were observed for all traits, and some of them were superior in comparison with the controls. The accumulated forage yield during three years was 988 g m^−2^ in the *P. notatum* hybrids, whereas, in *P. simplex*, the average forage yield per harvest (40 days of regrowth) was 180 g m^−2^. In *P. simplex*, the apomixis expressivity varied between 0 and 100%, and 65% of the hybrids showed high apomixis expressivity (superior to 70%). The genotypic mean homogeneity in the progeny was 76% and 85% in *P. notatum* and *P. simplex*, respectively. The generation of hybrids with high apomixis expressivity that combine good agronomic performance and homogeneity in the offspring is possible in tetraploid *P. notatum* and *P. simplex*.

## 1. Introduction

Grasslands represent 24% of the earth’s vegetation, and the Poaceae family is a fundamental component [1]. Grasslands of South America are a source of diversity for many forage species of the Poaceae family; in addition, they play an important role in the economy of many countries because they are the basis of livestock feed [2]. Thus, these ecosystems are important for conservation and as a source of diversity for the genetic improvement of forage species. The *Paspalum* genus has a large number of dominant species in tropical, subtropical, and temperate grasslands of The New World [3,4].

*Paspalum notatum* Flüggé and *Paspalum simplex* Morong are native to the Americas [5,6]. *P. notatum* is cultivated with different uses in the USA and several countries around the world [7]. It is a rhizomatous and grazing tolerant species, which is associated with light textured, well drained, and low fertility soils. Although *P. simplex* has been identified as an important forage component in native rangelands of semiarid regions [8], it is not cultivated mainly because of the lack of seed availability. It is an upright species associated with dry environments and fertile soils [9]. Furthermore, both species have different cytotypes, with the tetraploid being one of the most abundant in nature [5,10]. Additionally, the tetraploid cytotype reproduces by apomixis, which is an asexual mode of reproduction [6].

Apomixis allows for the cloning of genotypes through seeds [11]. This characteristic is interesting in plant breeding programs, since it ensures the same genotype through productive cycles. However, until now, it has not been possible to incorporate this trait into major crops. Furthermore, apomixis has been successfully explored and used in forage grasses, and several cultivars have been generated [7,12]. Breeding methods in apomictic and polyploid species are based on ecotype selection and hybridization. Ecotype selection consists of evaluating the greatest possible diversity of germplasm and selecting superior apomictic genotypes [13]. This method has been successful, since it makes it possible to obtain superior forage cultivars, such as *Brachiaria brizantha* cv. Marandu, which is the most cultivated forage in South America [12]. Another cultivar obtained by this method is *Megathyrsus maximus* cv Gatton, which was selected from a group of 13 *Megathyrsus* species collected around Africa [14]. The success of this method was related to the high diversity within species; however, it is limited by its inability to generate diversity and new genotypes that combine multiple traits [11]. Hence, hybridization appeared as an alternative to improve apomictic species after the development of sexual genotypes with the same ploidy level [13]. The purpose of this method is to release the natural diversity present in apomictic ecotypes and to fix superior apomictic F_1_ hybrids. The cultivars Mulato and Mulato II from *Brachiaria* were developed using this technique. Mulato is the result of crosses between *Brachiaria ruziziensis* × *B. brizantha* cv. Marandu, while Mulato II is the product of combining *B. ruziziensis*, *Brachiaria decumbens* cv. Basilisk, and *B. brizantha* [15,16].

In the genus *Paspalum*, ecotype selection has been the most widely used breeding method that has made it possible to release a vast number of forage cultivars for *Paspalum atratum* Swallen, *Paspalum dilatatum* Poir., *Paspalum genoarum* Arechav., *Paspalum nicorae* Parodi, *P. notatum*, and *Paspalum plicatulum* Michx. [6]. After the generation of sexual tetraploid genotypes, hybridization started to play a more important role in *Paspalum* breeding. Many F_1_ hybrids with forage potential have been generated in different species of the genus [17,18,19,20,21,22], however, just one of them, *P. notatum* cv. Boyero UNNE, was released to the market [5].

The generation of sexual tetraploid plants was possible in *P. notatum* and *P. simplex* by doubling the chromosome number of sexual diploid plants [23,24,25,26]. These induced tetraploid plants were crossed with diverse apomictic tetraploid genotypes, which were collected from different sites and preserved in a germplasm bank located in Corrientes, Argentina, and large segregating populations were obtained [17,18,19]. The mode of reproduction of the resulting hybrids was variable and dependent on the combination of progenitors. In *P. notatum*, Zilli et al. [19] reported an overall average segregation ratio between sexual and apomictic hybrids of 3.2:1, which ranged from 1:1 to 7.4:1 among families. According to Brugnoli et al. [18], *P. simplex* showed an average segregation ratio between sexual and apomictic hybrids of 2.4:1, which ranged from 0.6:1 to 6.5:1 among families.

The apospory expressivity of the apomictic hybrids of *P. notatum* was evaluated by the observation of mature embryo sacs [19]. It was variable (1–100%), and 32% of the apomictic hybrids showed high values of apospory expressivity (81–100% expressivity). It would be of great interest to analyze this trait in *P. simplex* to determine if the pattern observed for *P. notatum* is common in the genus. In this species, the aposporic and meiotically derived embryo sacs contain antipodals that make it difficult to diagnose the mode of reproduction by microscopic observations [27]. Hence, other methods, such as the flow cytometric seed screen [28], can be used to estimate the expressivity of apomixis.

The breeding process of apomictic species also involves the evaluation of the performance of the promising apomictic hybrids. In the case of *P. notatum*, Zilli et al. [19] identified a group of highly apomictic hybrids that exhibited good forage yields, cold tolerance, season regrowth, and seed yields. However, those hybrids were evaluated as individual plants during one growing season, so it would be important to test them at a greater scale and for more than one growing season. In *P. simplex*, Brugnoli et al. [18] generated a group of apomictic hybrids, which have not been evaluated for the level of apomixis expression. Additionally, the seasonal growth of those hybrids was estimated; however, forage yield evaluations at a greater scale than that of individual plants were not conducted. According to the given findings, it would be of great interest to determine the apomixis expression levels of the selected *P. simplex* hybrids and evaluate their forage yields, since this species has the potential to be cultivated in the subtropics.

As was mentioned above, the goal in the breeding of apomictic species is to generate superior highly apomictic hybrids. In this way, the superior characteristics will stay stable through growing cycles. The expressivity of the mode of reproduction can be evaluated at two moments: at early stages of the flowering phase or in the progeny [5,19]. The first option represents an advantage over the second in terms of time and resources. However, estimating the expression of apomixis in the progeny makes it possible to determine the offspring homogeneity of an apomictic hybrid, which is an important attribute in a future new apomictic cultivar [5]. In addition, studying the expressivity of apomixis at both stages would make it possible to determine if the levels of apomixis are conserved at flowering and within the resulting pasture. It would be of great interest to determine the expression of the apomixis in the progenies of the novel apomictic hybrids of *P. notatum* and *P. simplex* and determine their offspring genotypic variability. 

The objectives of this research were (1) to evaluate a group of novel apomictic hybrids of *P. notatum* and *P. simplex* for a set of agronomic traits in plots, (2) to estimate the apomixis expressivity in seeds of *P. simplex* apomictic hybrids, and (3) to determine the genotypic homogeneity of the progeny of *P. simplex* and *P. notatum* apomictic hybrids.

## 2. Materials and Methods

### 2.1. Plant Material and Agronomic Evaluation

#### 2.1.1. *P. notatum*

In this research, each F_1_ genotype resulting from a cross was named as a hybrid. This study comprised 13 apomictic hybrids of *P. notatum* characterized as highly apomictic and superior for a series of agronomic traits [19] (Table 1), including 4 apomictic hybrids from the University of Florida [17], 2 cultivars of *P. notatum* (cv. Argentine and cv. Boyero UNNE) used as intraspecific controls, and *Chloris gayana* cv. Callide as an interspecific control. This last species was chosen because it is cultivated in the same environment as *P. notatum*. Two hundred seeds from each genotype were sown in trays with potting mix in the greenhouse in September 2015. One hundred seedlings of each genotype were transplanted into seedling flats in October 2015. Three-tiller plants were planted in a field, near the city of Corrientes (27°28′45″ S, 58°47′06″ W), into 1.2 × 1.2 m plots, with 25 plants in each plot spaced 30 cm between them. The soil type was classified as Argiudoll. The experimental design was a randomized complete block with 3 replicates.

Three agronomic traits were evaluated: forage yield, seed production, and cold tolerance. Forage yield was measured during three growing periods (2015–2016, 2016–2017 and 2017–2018) by cutting the plots at 10 cm stubble height. Four to five harvests per year were made. The fresh weight of the harvested forage mass was recorded, and a subsample was collected and dried at 60 °C for 48 h. The dry subsample was weighed (g), and the amount of harvested biomass was calculated.

Pure seed production was measured in February 2016. Inflorescences were hand harvested, dried, manually threshed, and cleaned using a seed blower. It is important to clarify that no significant difference was previously observed for seed weight among genotype (100-seed weight ≅ 0.33 g). Cold tolerance was estimated in July 2017, two days after a frost event, using a 1-to-5 visual scale, where 1 represented plants exhibiting the least tolerance, and 5 represented the greatest tolerance.

The software Info-Stat [31] linked to R Studio platform was used to fit a mixed linear model where genotype and harvests and their interaction were considered fixed effects, whereas blocks were considered as random effect, constant correlation within plots, and heteroscedastic errors. Comparisons between two means were performed using the LSD test at *p* = 0.05, and comparisons between multiple means were performed using the Tuckey test at *p* = 0.05.

#### 2.1.2. *P. simplex*

The germplasm used for this study consisted of three apomictic ecotypes, with seven apomictic hybrids of *P. simplex* and *Megathyrsus maximun* cv. Gatton used as controls. The last species was chosen because is cultivated in the same environment where *P. simplex* grows. The ecotypes were collected from three different locations in Argentina, (Reconquista, Villa Ana, and Santa Ana), and were characterized by Brugnoli et al. [8]. Hybrids were previously obtained by crossing sexual tetraploid genotypes with apomictic tetraploid genotypes (Table 1) [18]. The three apomictic ecotypes were selected based on their diversity and forage yield [8]. The seven apomictic hybrids were selected by their good agronomic performance. Two hundred seeds of each ecotype and hybrid were sowed in the greenhouse during spring 2016 and were then planted in 1.5 × 1.5 m plots in a field located in Corrientes, Argentina (27°28′45″ S, 58°47′06″ W). Each plot consisted of twenty-five plants spaced 25 cm between them. The soil type was classified as Argiudol. The experimental design was a randomized complete block with three replicates. Plots were cut at 10 cm stubble height in August 2017. Forage yield and winter regrowth were evaluated. Forage yield was measured in October 2017 and February 2018 by cutting plots at 10 cm stubble height. The fresh weight of the harvested forage mass was recorded, and a subsample was collected and dried at 60 °C for 48 h. The dry subsample was weighed (g), and the amount of harvested biomass was calculated. Winter regrowth was estimated in September using a 1-to-5 visual scale, where 1 represented plants exhibiting the least regrowth, and 5 the greatest regrowth.

On the other hand, software Info-Stat [31] linked to R Studio platform was used to fit a mixed linear model where genotype and harvest and their interaction were considered fixed effects, whereas block was considered as random effect and independent homoscedastic errors. Comparisons between two means were performed using the LSD test at *p* = 0.05, and comparisons between multiple means were performed using the Tuckey test at *p* = 0.05.

### 2.2. Evaluation of Apomixis Expressivity in Seeds of P. simplex Hybrids

Apomixis expressivity was estimated using Flow Cytometric Seed Screen (FCSS) [28]. Seeds were collected from three apomictic hybrid families of *P. simplex* during Summer 2012. Each hybrid family consisted of 5 to 18 apomictic hybrids derived from crosses performed between sexual tetraploid plants and apomictic tetraploid (Table 1). Approximately 40 seeds from each hybrid were analyzed. Nuclei were isolated by chopping two seeds with a razor blade in 0.5 mL of nuclei isolation buffer (Partec P kit CyStain UV). Samples were incubated for 2 min and then filtered through a 30-µm nylon mesh directly into the sample tube, to which 1.5 mL of fluorescent stain 4a,6-diamidino2-phenylindole (DAPI) staining buffer (Partec P kit CyStain UV) was added. The mixture was incubated for 5 min at room temperature and analyzed with a Partec PA II (Ploidy Analyzer II) flow cytometer with the detector operating at 355 nm. At least 3000 nuclei were counted for each sample (two seeds). Data were analyzed using PA-II Partec FloMax software. The mean values of DNA content for embryo and endosperm were established to infer the sexual or apomictic origin of each seed.

The relative embryo/endosperm DNA content of a tetraploid seed was expected to be 4x/6x in a sexually formed seed, resulting from a 4x embryo (*n* + *n*) and a 6x endosperm (*n* + *n* + *n*). In contrast, a relative embryo/endosperm DNA content of 4x/10x was expected in seeds of apomictic origin, because the embryo in these seeds was formed by parthenogenesis of an unreduced egg cell (2*n* + 0), whereas the endosperm arose from pseudogamy that involved the central cell (carrying two unreduced polar nuclei) fertilized by one reduced sperm cell of the pollen tube (2*n* + 2*n* + *n*). These embryo/endosperm ratios of DNA content in sexual and apomictic plants were previously determined for *P. simplex* by Galdeano et al. [32].

The expressivity of apomixis was determined as the proportion of seed originated by apomixis.

### 2.3. Genotypic Homogeneity within the Progeny

A progeny test based on molecular markers of Inter-Simple Sequence Repeat (ISSR) was used to estimate the genotypic variability within the progeny of each hybrid. The proportion of identic genotypes of the progeny to the mother plant (genotypic homogeneity) indicate the expressivity of apomixis of a given hybrid. Hybrids used were previously identified as highly aposporic (expressivity higher than 70%) by the observation of mature embryo sacs in *P. notatum* and FCSS in *P. simplex*. One hundred seeds of each of the 7 *P. notatum* and 6 *P. simplex* hybrids were sown in September 2014. Twenty-five seedlings for each hybrid were transplanted to pots. Young leaves were used for DNA genomic extraction using the same protocol descripted by Brugnoli et al. [8]. Genomic DNA was quantified by visual comparison with a known concentration DNA pattern, using electrophoresis in agarose 1% (*w*/*v*) gels in 1X TAE buffer (40 mM Tris-HCl, 5 mM sodium acetate, 0.77 mM EDTA, pH 8.0) at 40 V for 2 h. Genomic DNA was visualized under ultraviolet (UV) light and photographed with GelDoc-It Imaging System (UVP LLC) after staining with ethidium bromide (10 µg mL^−1^). Each DNA sample was adjusted to 10 ng μL^−1^ for their use in polymerase chain reaction (PCR) amplifications. Between 5 and 6 primers of ISSR were used for PCR amplifications. The ISSR markers were generated according to the methodology described by Zilli et al. [19]. Polymerase chain reaction products were separated by electrophoresis in 2% agarose gels in 1X TAE buffer, at 70 V for 3 h and stained with ethidium bromide (10 µg mL^−1^). The molecular profiles were visualized under UV light, photographed, and stored for further analysis with GelDoc-It Imaging System.

Inter-simple sequence repeat products were scored for the presence (1) and absence (0) of homologous DNA bands. Molecular diversity between progenies and mother was estimated with the number of polymorphic loci using the GenAlEx 6 program and the proportion of different genotypes of progenies and plant mother using the GenoType and GenoDive software [33].

The apomixis expressivity was calculated as the percentage of progeny identical to their female progenitor. The genotypic homogeneity of the progeny was estimated based on the variability observed within the progeny of each hybrid.

## 3. Results

### 3.1. Agronomic Evaluations

#### 3.1.1. *P. notatum*

When forage yield was evaluated, the mixed model analysis revealed no significant differences (*p* > 0.05) among genotypes, significant differences among months of harvest (*p* < 0.01) and significant interactions between genotype and month of harvest (*p* < 0.05). Due to the interactions found, it was decided to show the means comparison per month of harvest (Table 2). During the first and second year, no significant differences were observed among the *P. notatum* genotypes, including the 17 new hybrids, Argentine, and Boyero (Table 2). Regarding the third year, significant differences among *P. notatum* genotypes were observed in September and February (Table 2). The hybrids B7 and UF13 exhibited the highest values in September, while the hybrids C11 and C20 exhibited the highest values in February. The accumulated forage yield was not significantly different among genotypes during the three evaluated years (Table 2). The mean accumulated forage yield of the new hybrids during the first, second, and third year were 1010 g m^−2^, 1037 g m^−2^, and 916 g m^−2^, respectively. The forage yield of *P. notatum* was less than *C. gayana* cv. Callide during the first two years. However, all *P. notatum* hybrids exhibited greater values than Callide in the third year (Table 2).

The seed yield was highly variable among hybrids, with a range from 20 to 240 kg ha^−2^ (Table 2). The genotypes I7, Argentine, UF67, and UF13 exhibited the highest values. No seed was harvested from *C. gayana* cv. Callide, because the stand of plants was severely affected by the frequency of defoliation during the three years. Cold tolerance was also variable, with UF13 being the most tolerant genotype, followed by I7, C3, UF3, and UF93 (Table 2). The less tolerant genotypes were F44, I21, B7, and Callide (Table 2).

#### 3.1.2. *P. simplex*

The forage yield and winter regrowth of a group of *P. simplex* hybrids and ecotypes, as well as *M. maximus* cv. Gatton, were evaluated. The mixed model analysis revealed significant differences (*p* < 0.05) among genotypes for forage yield, no significant difference among month of harvest (*p* > 0.05), and no significant interactions between genotype and month of harvest (*p* > 0.05). Thus, it was decided to show the means of two harvests per genotype (Table 3) Hybrid mean forage yields were 179 gm^−2^ and 226 gm^−2^ for ecotypes. The highest forage yield between ecotypes was exhibited by the ecotype from Reconquista, while, among hybrids, the highest forage yield was from the hybrid A26. In addition, the same two genotypes exceeded the forage yield of the interspecific control (Table 3). Regarding winter regrowth, it was variable among genotypes (*p* = 0.0001) with one group having the greatest regrowth (Villa Ana, A26, and A 27) and another having the lowest regrowth (Santa Ana, A19 and A12) (Table 3).

### 3.2. Apomixis Expressivity in P. simplex

**Figure 1:**
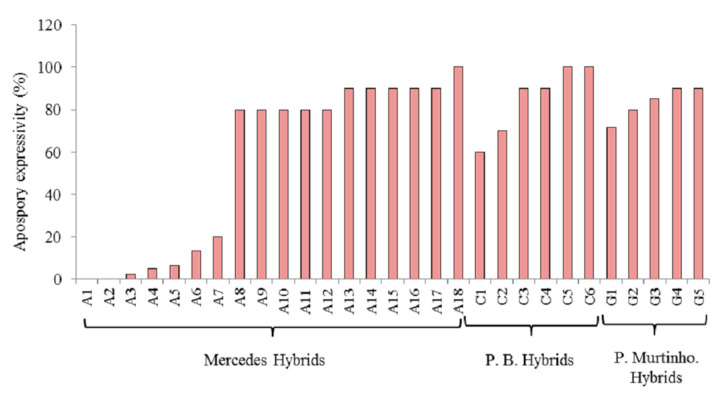
Apomixis expressivity of three families of hybrids of *P. simplex* analyzed by flow cytometry. P.B: Piedras Blancas hybrids.

**Figure 2:**
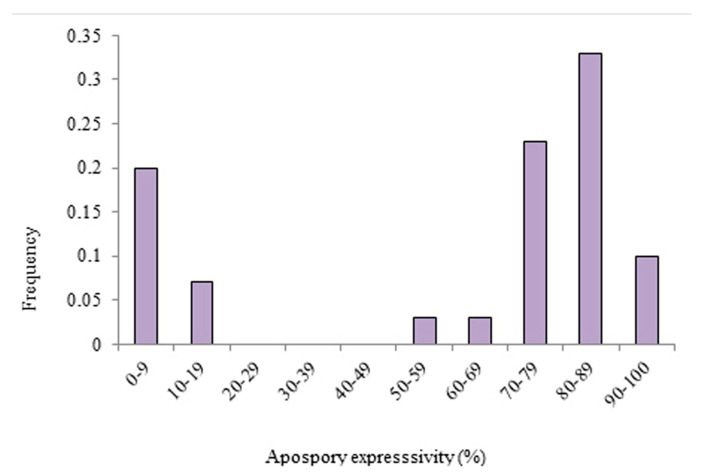
Level of apomixis expressivity for a group of 30 tetraploid apomictic hybrids from 3 different sexual x apomictic *P. simplex* families.

The apomixis expressivity of a group of 30 hybrids of *P. simplex* that were previously classified as apomictic by Brugnoli et. al. [18] was evaluated using a flow cytometric seed screen (Figure 1). Apomixis expressivity was different between families. The expressivity of the hybrids of the Mercedes family was between 0 and 100 percent, while the hybrids belonging to the Piedras Blancas and Porto Murtinho families only showed high levels (between 60 and 100%) (Figure 1). When all hybrids were evaluated for apomixis expressivity, two groups were identified in total (Figure 2). One group was characterized by low levels, between 0 and 20%, and another by medium to high levels, between 50 to 100%. Levels of apomixis expressivity between 20% and 50% were not observed. In addition, the fraction of hybrids with high expressivity (greater than 80%) was 65% (Figure 2).

### 3.3. Genotypic Homogeneity in Progenies of P. notatum and P. simplex Hybrids

**Figure 3:**
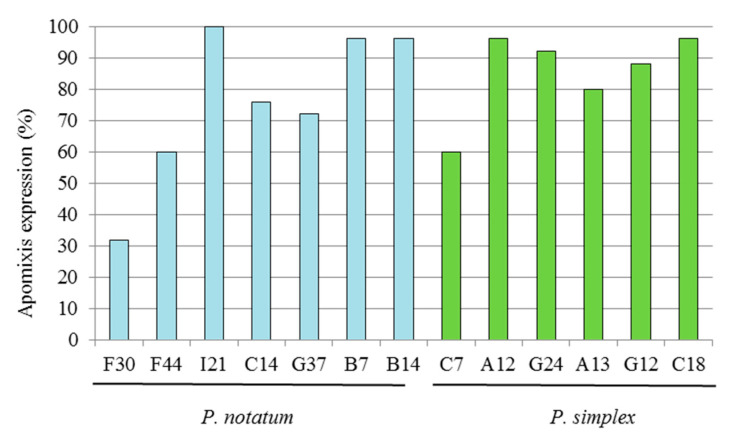
Expression of the apomixis (%) in the progeny of a group of highly apomictic hybrids from *P. notatum* and *P. simplex*.

The genotypic homogeneity was estimated using progeny tests for a group of seven hybrids of *P. notatum* and six hybrids of *P. simplex*. In total, 181 genotypes of *P. notatum* and 155 of *P. simplex* were evaluated using ISSR markers. A total of 157 and 119 ISSR bands were evaluated for *P. notatum* and *P. simplex*, respectively. Hybrids of *P. notatum* and *P. simplex* selected for this experiment showed high expressivities of apomixis and high homogeneities within the progeny (more than 70%). In *P. notatum*, high apomixis expressivity and progeny homogeneity (greater than 70%) was observed on average, except for hybrids F30 and F44 that exhibited a lower value (32 and 60%, respectively) (Figure 3). In the case of *P. simplex*, 5 out of 6 hybrids had expression levels greater than 80%, and only the hybrid C7 showed a lower expression (60%).

## 4. Discussion

The main objective of the breeding programs of apomictic species by hybridization was to develop highly apomictic hybrids with superior agronomic traits, since this mode of reproduction makes it possible to fix superior characteristics through growing cycles [11]. One important step in the breeding process involves the evaluation of the performance of the hybrids. Hence, in this work, new intraspecific apomictic hybrids of *P. notatum* and *P. simplex* and a group of intraspecific and interspecific controls were intensively evaluated for agronomic characteristics. The forage yields of *P. notatum* hybrids were similar to the intraspecific controls during the first and second year of evaluations. This result could be related to the high selection pressure performed over the hybrids at the individual plant stage evaluation [19], thus resulting in hybrids producing as much forage as the cultivars used as controls. In addition, in this work, the combination of parents was defined to cover as much genotypic diversity as possible; however, a more efficient selection of the best parents could be used to obtain better hybrids [34]. Regarding cold tolerance, some of the new hybrids exhibited high values, and they were able to grow during the cold season, which is a highly sought characteristic in perennial warm-season grasses. Seed yields among hybrids ranged from 20 to 240 kg ha^−1^. Hybrid I7 showed the greatest seed yield, consequently over-yielding the intraspecific cultivars used as controls. This characteristic is necessary for the seed industry to ensure seed availability at an appropriate cost, thus enabling the adoption of new cultivars. Forage yields of *P. notatum* hybrids were lower than *C. gayana* cv. Callide during the first two growing periods, while in the third period, some hybrids exhibited greater values than the interspecific control. This could be related to the time *P. notatum* required to be well established. Additionally, the growth of *P. notatum* was less affected over time, which might be related to its prostrate growth habit. Furthermore, the *P. notatum* apomictic hybrid UF13 exhibited greater cold tolerance than *C. gayana* cv. Callide. Contrastingly, the forage yields of the new *P. simplex* hybrids were variable and not affected by the year of harvest. One hybrid exceeded the forage yields of a group of selected apomictic ecotypes of the species and of the interspecific cultivar used as control (*M. maximus* cv. Gatton). Regarding winter regrowth, one of the new hybrids exhibited one of the best values alongside one ecotype. These results show the forage potential of the new apomictic hybrids of both *Paspalum* species. Previous works conducted in the genus revealed that it is possible to develop superior apomictic hybrids for agronomic traits [17,18,19,20,22,35]. However, these studies were carried out using individual plants and during one or two growing periods, while, in this work, the evaluations were conducted at a greater scale and in three growing periods, in the case of *P. notatum*.

The generation of hybrids in the *Paspalum* genus was carried out on several occasions and species in order to achieve heterotic hybrids with high expressiveness of apomixis. Variable levels of apomixis expressivity were observed in *M. maximus* [36] and *P. notatum* [17,19,34] apomictic hybrids. This phenomenon is still unclear, but it may be a consequence of various factors, such as the genetic or epigenetic background, ploidy levels, as well as environmental factors [6,19,35,37,38]. The apospory expressivity of the *P. notatum* hybrids used in this work was previously evaluated by Zilli et al. [19]. The authors found that most hybrids exhibited a low or high apospory expressivity, but a reduced proportion of them exhibited an intermediate level. In the case of *P. simplex*, this is the first report of the range of variation for the expressivity of apomixis in the species. Highly apomictic hybrids were detected, and a similar discontinuous variation as in *P. notatum* was observed. These results indicate that, in both species, it is possible to develop highly apomictic hybrids. In addition, the discontinuous variation of the trait may be considered as a common pattern in the genus *Paspalum*, since two species belonging to two taxonomic groups within the genus exhibited the same pattern.

A high level of apomixis expressivity is the key for the release of new hybrid cultivars to the market, thus ensuring their homogeneity throughout reproductive cycles. However, in the *Paspalum* genus, the segregation pattern for the mode of reproduction in favor to the sexual offspring and the variable apomixis expressivity represent an obstacle to the breeding process. Hence, the analysis of the offspring genotypic homogeneity allows breeders to know how stable the apomictic hybrid is over time. Actually, it is possible to estimate the level of apospory expressivity (apomeiosis) at early stages of the reproductive phase through embryo sac observations [19]. The expressivity of apomixis (apomeiosis + parthenogenesis + endosperm development) can be measured in seeds by mean of FCSS [18,28,39] or in the progeny by DNA fingerprinting [31]. These methods of evaluation represent a great advance in terms of time and resources in comparison to the classic evaluation of morphologic traits on the offspring [6]. Although the DNA fingerprinting method takes place in the offspring and is time consuming, it represents an accurate way to determine the genotypic stability of a new hybrid. In *P. notatum*, two of the seven hybrids classified as highly aposporic by embryo sac observation exhibited a lower expression of apomixis at the progeny stage, while in *P. simplex*, just one of the hybrids showed a lower apomixis expression at the progeny stage in comparison to observation done at seed stage.

Variations in the expressivity of apomixis were observed throughout the reproductive stages in *P. notatum* [37], *Paspalum mallacophyllum* [40], *Paspalum maculosum*, and *Paspalum chromyorhizon* accessions [41]. In all these previous studies, embryo sacs observations or FCSS underestimated the level of apomixis expressivity observed with a progeny test. The results obtained in this work do not show the variation in expressivity at different stages as reported in other species of the genus. This could be related to the kind of germplasm used, since, in this research, a group of new apomictic hybrids was evaluated, while, in the *Paspalum* species mentioned above, naturally occurring ecotypes were analyzed. Hybridization involves a recombination process between two genotypes, while, in the naturally occurring ones, this event does not take place. Taking into account all the above-mentioned factors, the use of molecular markers linked to apospory, embryo sac observation, and/or FCSS to estimate apospory or apomixis expressivity are valuable technologies that allow breeders to save time and resources. However, in advanced selection stages, other techniques, such as DNA fingerprinting, would be necessary to characterize the stability of apomixis at the progeny stage in promising lines. In addition, further research is needed regarding genetic, epigenetic, and environmental factors affecting apomixis expression in order to improve the breeding efficiency, as well as to develop management practices for the seed industry of apomictic cultivars aimed to obtain homogeneous pasture stands. A uniform progeny is a fundamental attribute in an apomictic cultivar, since this ensure its reproducibility [5]. In this work, most of the highly apomictic hybrids exhibited high levels of apomixis (greater than 70%) in the offspring. This means that the progeny of those hybrids was highly uniform and that their superior characteristics will persist over different growing cycles. All these indicate that, in *P. notatum* and *P. simplex*, it is possible to generate uniform and reproducible forage hybrids.

In conclusion, the tetraploid germplasm of *Paspalum* can be used to generate superior forage cultivars by hybridization and fixation via apomixis of the superior progeny. A similar pattern of apomixis expressivity was observed within the progeny for two unrelated species of *Paspalum*. There was a strong relationship between the apomixis expressivity observed at flower or seed stages and the resulting progeny, thus early diagnosis should be considered effective. The results obtained in this research indicate that, in *P. notatum* and *P. simplex* hybrids, the high levels of apomixis stay stable over time, and that it is possible to combine this high apomixis expressivity with good forage attributes in a single genetic line. A more efficient selection method can be used for the selection of heterotic parents to generate better hybrids as part of the next phase of the breeding program.

## Figures and Tables

**Table 1:** Identification and origin of hybrids of *Paspalum notatum* and *P. simplex* used in this experiment.

Species	Female Sexual Parent	Male Apomictic Parents	Apomictic Hybrid Family
*P. notatum*	SWSB (sexual white stigma bahiagrass, derived from hybrids originally generated by G.W. Burton by crossing an induced tetraploid plant with an apomictic natural tetraploid bahiagrass with white stigmata).	Q4064 (ecotype from Saladas, Corrientes, Argentina)	B [18]
	Q4205 (obtained by self-pollination of SWSB)	Q3776 (ecotype Villa Tunari, Chapare region, Bolivia)	G [18]
	Q4205	Q4064	I [19]
*P. simplex*	C1-2 (self-pollination of a colchicine-induced autotetraploid plant obtained from the Istituto di Ricerche sul Miglioramento Genetico delle Piante Foraggere del Consiglio Nazionale delle Ricerche, Perugia, Italy) [25,29]	Mercedes (ecotype from 29°10′ S, 58°04′ W)	A [18]
	C1-2	Piedras Blancas (ecotype from 31°11′ S, 59°57′ W)	C [18]
	C1B2 (self-pollination of a colchicine-induced autotetraploid plant obtained from the Istituto di Ricerche sul Miglioramento Genetico delle Piante Foraggere del Consiglio Nazionale delle Ricerche, Perugia, Italy [25,30]	Porto Murtinho (ecotype from 21°42′ S, 57°51′ W)	G [18]

**Table 2:** Forage yield (g m^−2^), seed yield (kg.ha^−1^) and cold tolerance of a group of *Paspalum notatum* hybrids, cv Argentine, cv Boyero UNNE, and *C. gayana* cv Callide during three growing periods in Corrientes, Argentina.

		Forage Yield First Year						Forage Yield Second Year					Forage Yield Third Year					Seed Yield	Cold Tolerance *
		Febr	March	May	Sep	Dec	Accu	Febr	March	June	Nov	Accu	Febr	June	Sep	Febr	Accu		
Controls	cv. Argentine	166	165	74	31	264	700	205	271	299	257	1032	202	252	16	198	668	200	3
	cv. Boyero UNNE	274	215	148	86	268	991	282	261	304	292	1139	272	301	64	276	913	100	2.3
	cv. Callide	378	379	270	114	503	1644	59	350	304	137	850	225	197	nd	285	707	nd	1
*P. notatutm hybrids*	F44	260	257	146	86	219	968	269	259	223	240	991	259	280	41	244	824	80	1
	I21	223	226	119	56	217	841	164	230	235	258	887	230	301	43	271	845	90	2.3
	I7	286	274	128	39	377	1104	273	262	232	204	971	269	330	9	273	881	240	3.3
	C11	266	268	152	97	369	1152	120	264	254	361	999	335	360	43	425	1163	50	2
	C14	276	276	184	93	343	1172	367	342	272	294	1275	384	360	70	280	1094	70	2
	C20	215	211	358	73	224	1081	169	253	254	162	838	251	281	34	448	1014	40	2.3
	C21	212	219	94	67	173	765	191	169	208	202	770	354	375	52	271	1052	50	1.3
	C3	270	279	113	75	248	985	279	270	248	328	1125	267	347	53	358	1025	120	3.3
	G37	252	255	148	63	359	1077	281	369	314	363	1327	321	352	31	283	987	50	1.7
	G6	220	214	69	39	245	787	212	235	199	220	866	163	238	48	261	710	50	3
	B12	203	171	96	71	194	735	169	186	209	332	896	166	231	57	262	716	20	2.3
	B14	149	171	109	49	305	783	212	237	259	281	989	199	280	38	368	885	60	3
	B7	314	334	243	119	356	1366	330	313	323	367	1333	264	339	81	309	993	20	1
	UF13	247	220	182	99	481	1229	322	279	246	380	1227	377	283	78	223	961	150	4.7
	UF3	212	215	190	107	355	1079	276	270	198	287	1031	337	286	52	244	919	70	3.3
	UF67	242	228	130	46	357	1003	226	271	244	176	917	268	197	24	195	684	160	2.3
	UF93	225	221	130	47	416	1039	302	282	267	340	1191	294	254	40	230	818	70	3.3
	MSD	182	182	375	144	418	1029	324	237	190	258	844	389	323	58	222	745	110	2.9

MSD: Minimum significant difference at 5% by the Tukey test, nd: no data. * 1 to 5 visual scale, where 1 represented plants exhibiting the lowest tolerance and 5 represented the greatest tolerance. Febr: February; Sep: September; Nov: November; Dec: December, and Accu: accumulated forage yield.

**Table 3:** Forage yield (g m^−2^) of a group of *P. simplex* hybrids and ecotypes, and *M. maximus* cv. Gatton in Corrientes, Argentina.

	ID	Forage Yield	Winter Regrowth *
*Control*	*M. maximus* cv. Gatton	166.0	3.4
*P. simplex* ecotype	Reconquista	244.5	3.7
	Villa Ana	217.7	4.7
	Santa Ana	215.5	1
*P. simplex* hybrids	A26	250.5	4.3
	C 24	234.5	3.7
	A 27	232.0	4.0
	A 19	177.6	1.3
	A 14	168.4	3.7
	A17	103.1	2.3
	A12	91.5	1.3
	MSD	130	1.7

MSD: minimum significant difference at 5% by the Tukey test. * 1-to-5 visual scale, where 1 represents plants exhibiting the lowest regrowth, and 5 the greatest regrowth.

## Data Availability

Not applicable.
